# Supplementary material for: Trend and determinants of minimum dietary diversity consumption change among children aged 6–23 months in Ethiopia from 2011 to 2019: A multivariate decomposition analysis
Source: PLoS One. 2024 Aug 2;19(8):e0308349. doi: 10.1371/journal.pone.0308349 (PMC11296628; doi:10.1371/journal.pone.0308349)
Supplement: Attachment — Submitted filename: Authors Response to Reviewers.docx [file pone.0308349.s002.docx]

**Authors’ Responses to Editor and Reviewers Comments**

**Dear Academic Editor, Plose One**

“**Trend and Determinants of Minimum Dietary Diversity Consumption Change among Children Aged 6-23 months in Ethiopia From 2011 to 2019: A Multivariate Decomposition Analysis**”

Dear Plos One Editors and Reviewers;

We are thankful for your constructive comments. We have looked at the comments and have revised our paper accordingly. We hope our paper improved as a result of incorporating the reviewer’s and academic editor’s comments and suggestions. Here are the authors’ responses to the comments.

Please find for your kind consideration the following:

1. A revised manuscript with track changes.
2. A revised paper without tracked changes
3. A rebuttal letter that responds to each point raised by the academic editor and reviewer.

The point-by-point responses of authors are written by hoping these changes would meet with your favorable consideration, we are happy to hear if there are more comments and suggestions. Please do not hesitate to let us know if you have any questions.

Yours Sincerely

Mr. Abel Endawkie Correspondence Author

Department of Epidemiology and Biostatistics School of Public Health College of Medicine and Health Science Wollo University Dessie Ethiopia

Tel. 251935459310	Email address abelendawkie@gmail.com

We have tried our best to improve it accordingly:

Please revise the manuscript.

Journal requirements:

When submitting your revision, we need you to address these additional requirements.

1. Please ensure that your manuscript meets PLOS ONE's style requirements, including those for file naming. The PLOS ONE style templates can be found at

<https://journals.plos.org/plosone/s/file?id=wjVg/PLOSOne_formatting_sample_main_body.pdf> and<https://journals.plos.org/plosone/s/file?id=ba62/PLOSOne_formatting_sample_title_authors_affiliations.pdf>

**Response 1:** We revised our manuscript based on Plos one submission guidelines (manuscript, title format, and table format).

**Point-by-point response of Authors for editors' and reviewers' comment**
**Editor's Comments to the Authors**

Additional Editor Comments:

The authors are requested to address the reviewers' comments carefully and in detail

**Response:** we are grateful to thank you for your kindness and response. We tried to correct it thoroughly.

**Reviewer Comment to the Authors**

**Reviewer#2 comment: Thank you for responding to the comments raised. However, you did not respond to whether to accounted for collinearity. Also, I fail to see the theoretical perspective guiding this research. Why so?**

**Response:** Thank you for your feedback and for taking the time to review the comments. I apologize for any inconvenience caused by my previous response. Regarding the issue of collinearity, I would like to assure you that it has been duly accounted for in our research. In terms of the theoretical perspective guiding our research, we have indeed considered this aspect. The theoretical framework of our study is grounded in an insert theoretical framework, which provides a foundation for understanding the phenomena under investigation. We have thoroughly reviewed the relevant literature and integrated existing theories to inform our research design and analysis.

**The response is provided below in detail.**

**Comment** 1: Did you account for collinearity?

**Response 1:** First and foremost, we express our sincere gratitude for your intriguing idea and thoughtful questions. We would like to sorry for this inconvenience. The inconvenience happened due to we did not report the variable selection process and multicollinearity assessment as we believe it was long even it affected our transparency and reproducibility for the scientific work. We value your insights and suggestion since Multicollinearity is a problem when independent variables are correlated and analyzed in the model.

As we know Multicollinearity occurs when multiple independent variables in a regression model are highly correlated with each other and Multivariate decomposition analysis, aims to partition differences in mean responses between groups or over time into components that reflect both the differences in mean levels of model predictors and the differences in the effects of those predictors across groups or periods.

Even though these two concepts address different aspects of statistical analysis, they can be combined to understand the impact of multicollinearity on the decomposition of group differences. The way to detect multicollinearity in multivariate decomposition differs from the usual work of other regression models.

Detecting Multicollinearity:

The most common way to detect multicollinearity is by using the variance inflation factor (VIF). VIF measures the correlation and strength of correlation between predictor variables in a linear regression model. High VIF values (typically above 5 or 10) indicate strong multicollinearity

Multicollinearity was checked among explanatory variables by using average standard error at a cut of point±2 for the logistic model.

Because Multicollinearity detection in multivariate decomposition is before performing multivariate decomposition, we simply screened the data whether it has Multicollinearity or not using the logistic model before we conducted multivariate decomposition because the decomposition is multivariate decomposition for the logit model. Not only this the variable selection was conducted before multivariate decomposition analysis using Bivariable logistic regression of p-value <0.25.

In our analysis, we assessed Multicollinearity using the average standard error of each standard error since we had a binary outcome variable and we used logistic regression.

Since it is important we shortly wrote the variable selection process and multicollinearity assessment to develop a multivariate decomposition analysis in the method section as below.

Before analysis first, bi-variable logistic regression was fitted and variables with p-values less than 0.25 were selected to develop multivariate decomposition analysis. Multicollinearity was checked among explanatory variables by using average standard error at a cut of point±2 and there is no Multicollinearity since the standard errors were between±2.

The observed procedure arises due to the inherent nature of multivariate decomposition analysis applied to a nonlinear (logit) model.

**Comment 2:** Given the nature of this study, it will be advisable for the authors to include a theoretical framework to strengthen their findings and lay the foundation for their arguments in the discussion section

**Response 2:** First and foremost, we express our sincere gratitude for your intriguing ideas and thoughtful questions. We would like to sorry for this inconvenience. As you suggest the theoretical framework helps to strengthen the findings and discussion:

In the context of child feeding practices and MDD consumption, the framework suggests that the trend of MDD consumption change is determined by the change in different factors: including composition change at the individual level like child characteristics (age, sex), maternal characteristics (age, education, occupation), composition change at the household characteristics (wealth status, household head sex, number of under-five living children, and household size), composition change at the community-level factors: residence, region, and behavioral change (coefficient) (figure 1). Then the trends and determinants of MDD consumption change found in this study can be interpreted through the lens of this theoretical framework that changes in individual, household, and community-level factors over time likely contributed to the observed trends in MDD consumption change. The conceptual framework was developed using literature.

The figure is provided next to the idea of the paragraph based on the journal's guideline

**We appreciate your kind response and genuine communication. We hope these changes will meet your favorable consideration and we are happy to hear if there are more comments and suggestions. Please do not hesitate to let us know if you have any questions.**

**We feel all of you may understand our feelings and interests.**

**We are thankful for all!!**
